# The Mononuclear Phagocytic System. Generation of Diversity

**DOI:** 10.3389/fimmu.2019.01893

**Published:** 2019-08-09

**Authors:** Siamon Gordon, Annette Plüddemann

**Affiliations:** ^1^College of Medicine, Graduate Institute of Biomedical Sciences, Chang Gung University, Taoyuan City, Taiwan; ^2^Sir William Dunn School of Pathology, University of Oxford, Oxford, United Kingdom; ^3^Nuffield Department of Primary Care Health Sciences, University of Oxford, Oxford, United Kingdom

**Keywords:** mononuclear phagocyte, macrophage, tissue-specific function, monocyte, plasticity, macrophage heterogeneity, macrophage receptors

## Abstract

We are living through an unprecedented accumulation of data on gene expression by macrophages, reflecting their origin, distribution, and localization within all organs of the body. While the extensive heterogeneity of the cells of the mononuclear phagocyte system is evident, the functional significance of their diversity remains incomplete, nor is the mechanism of diversification understood. In this essay we review some of the implications of what we know, and draw attention to issues to be clarified in further research, taking advantage of the powerful genetic, cellular, and molecular tools now available. Our thesis is that macrophage specialization and functions go far beyond immunobiology, while remaining an essential contributor to innate as well as adaptive immunity.

## Introduction

**Figure 1:**
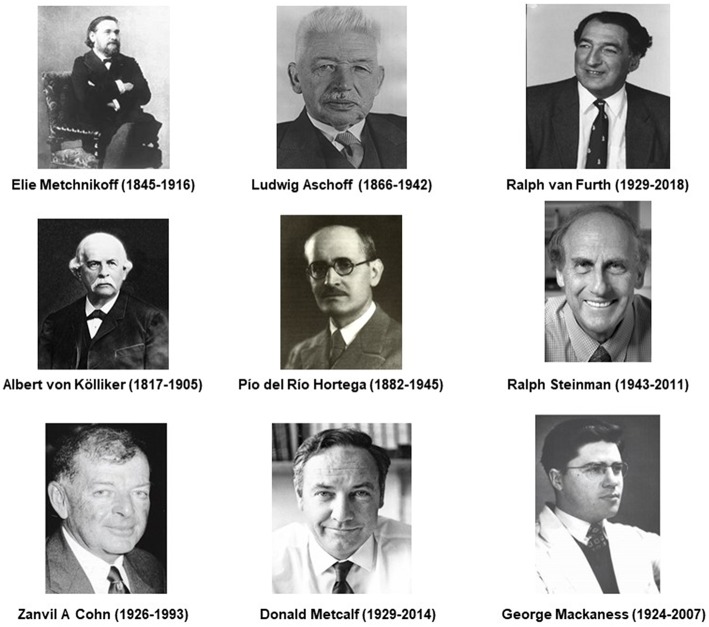
Historic figures associated with macrophages, related cells and their specialized functions.

Participation in several Ceppellini workshops by one of the authors (SG) provided an opportunity to examine and present to young investigators some aspects of the unique features of the macrophage, a cell type with an ancient origin in eukaryotic evolution. SG's attachment to the macrophage family has extended over 50 years, rejuvenated over every decade as methodological advances brought new insights and information. However, their biological role in the multicellular organism has remained incomplete, eclipsed as accessory to the specific recognition, and effector functions of lymphoid cells. Metchnikoff already appreciated their professional phagocytic capacity, their digestive proficiency, and potential role in antimicrobial defense (1), while Ehrlich and Wright (2) drew attention to the role of antibodies and opsonins, which enhance phagocytic uptake by monocytes, macrophages, and polymorphonuclear neutrophils (PMN). The discovery of complement and, decades later, the plasma membrane receptors for the Fc domain of IgG specific antibodies and for C3 activated by the classical and alternative pathways, initiated pioneering studies by many investigators [reviewed by Taylor et al. (3)]. Hortega recognized the special properties of microglia in the Central Nervous System (CNS) (4). The discovery of Dendritic cells(DC) by Steinman and Cohn (5, 6), demonstrated their superior role in antigen capture, processing, and presentation to naïve lymphocytes of peptides, in association with the highly polymorphic Major Histocompatibility (MHC) antigens, thus inducing specific T and B lymphocyte activation and expansion (7). DC-like cells can be readily produced in culture of mouse bone marrow or human monocytes in Granulocyte Macrophage Colony-Stimulating Factor (GM-CSF; CSF-2) and IL-4 (8). To some extent DC eclipsed the role of macrophages in adaptive immunity, although their role in innate immunity was secured by the discovery of Toll-like Receptors (TLR) (9). The discovery and characterization of cytokines produced by and acting on macrophages, such as Tumor necrosis factor(TNF) (10) and IL-1 (11), prepared the way for anti-TNF therapy (12), to ameliorate destructive immunopathologies such as rheumatoid arthritis. Activation of macrophages by cellular immunity, characterized by Mackaness (13), was shown to be antigen dependent, but non-specific, and lead to the characterization of Interferon (IFN) gamma (14) as the sole mediator of classical activation produced by antigen-specific T lymphocytes and Natural Killer (NK) cells. After setting the stage above, further relevant milestones of macrophage history will be introduced in subsequent sections. Selected historic figures important in the present understanding of tissue macrophage diversity are shown in Figure 1.

## The Mononuclear Phagocyte System, a Dispersed Organ

Metchnikoff recognized migratory and sessile, fixed tissue phagocytic cells in his early studies of invertebrate development, by microscopy, and intravital labeling. Direct observation of their recruitment to foreign particles injected *in vivo* lead to further studies in many vertebrate species on their role in host defense against bacteria. Tissue macrophages were subsequently shown to be widely distributed as a system of related cells during development, in the adult steady state, during inflammation, and infection. Aschoff introduced the term Reticulo-Endothelial System (RES), hallmarked by the efficient clearance of particles from the circulation, and extravascular space (15). The imprecise RES nomenclature was replaced by that of the Mononuclear Phagocyte System (MPS) (16), to distinguish mononuclear monocytes and macrophages from PMN, while sharing their highly active capacity as phagocytes. Although widely used till the present day, this terminology is not perfect, since other cell types phagocytose dying cells, and some macrophage-related cells are poorly or even non-phagocytic (17). The diverse cells of the MPS cannot all be characterized by single antigen markers or unique functions expressed at all stages of cell differentiation or activation. Nevertheless, their origin and diversification have common features which point to the valid concept of a specific, dispersed myeloid lineage.

**Figure 2:**
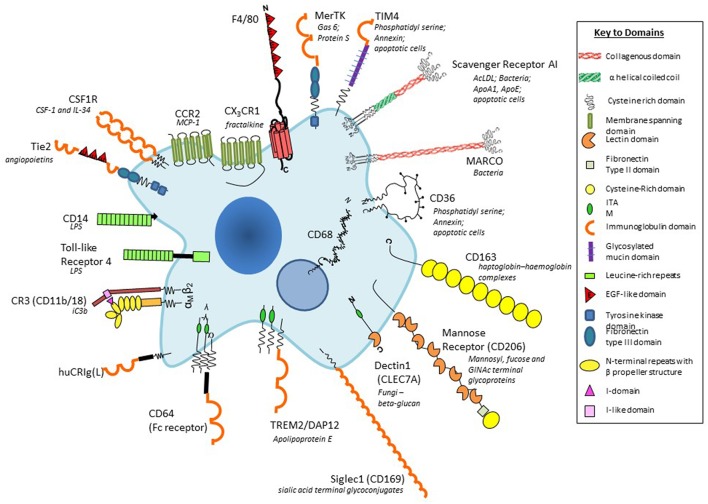
Plasma membrane antigens and receptors expressed by macrophages. Macrophages are able to express a large repertoire of membrane receptors implicated in the recognition and uptake of foreign and modified self-ligands, some of which are illustrated here. These receptors incorporate a range of structural domains, illustrated schematically; they serve as useful marker antigens for immunocytochemistry and FACS analysis (e.g., F4/80, CD68, CSF-1 receptor, Mer-TK, CD64). They function as opsonic (antibody and or complement coated particles to enhance uptake via Fc and complement receptors) or non-opsonic, carbohydrate-binding lectins, and scavenger receptors. The phagocytic receptors mediate clearance of microbes (e.g., MARCO), apoptotic cells (for example CD36, SR-A, TIM4), and circulating ligands; CCR2, and CX3CR1 are examples of GPCR receptors for the monocyte/macrophage chemokines MCP-1 and fractalkine, respectively; other receptors bind growth promoting and regulatory cytokines, for example, CSF-1, and angiopoietins (Tie-2), and CD163 for clearance of injurious haptoglobin–hemoglobin complexes. Toll-like receptor-4 and CD14 react with bacterial membrane components such as lipopolysaccharide (LPS) to induce pro-inflammatory signaling; Dectin-1 recognizes fungi through beta glucan in their wall, activating a range of innate immunological responses. Siglec-1 (CD169), a receptor for sialic acid terminal glycoconjugates, mediates adhesion of host cells and microbes, whereas CD206, a receptor for clearance of Mannosyl-, fucose-, GlcNAc-terminal glycoproteins, is a prototypical marker of M2-type activation. The scavenger receptor SR-A internalizes polyanionic ligands such as modified lipoproteins, as well as selected microbes, whereas CD36 mediates adhesion and M2-induced macrophage fusion and giant cell formation. TREM-2 mutations have been implicated in neurodegeneration and osteoclast function. For further details, see BMC, with permission (28).

During mammalian development, macrophages derive from haematopoietic precursors in para-aortic regions of the embryo, the yolk sac and fetal liver, seeding organs such as the brain and other tissues before birth (18, 19). A paradigm shift over recent decades has shown that after birth, in the absence of inflammation, resident macrophages in adult tissues derive from embryonic macrophages which can persist, and gradually turn over locally throughout adult life (20–22). This is especially the case for microglia in the (CNS) and Langerhans cells in the epidermis. The bone marrow, which develops as the main haematopoietic organ perinatally, and fumctions throughout adult life (23), serves to replenish resident tissue macrophages, for example in the gut (24), where macrophages turn over more actively, and provides blood monocytes (25) in response to increased demand, for example during inflammation and infection (26). The chemokines and receptors which mediate distribution of monocytes and macrophages in the fetus and adult are not completely defined, nor the adhesion molecules which determine organ-specific localization. Chemokines of resident macrophages include fractalkine and its receptor, CX3CR1 (27), and inflammatory, and immune monocyte recruitment mediated by CCL2 and its receptor, CCR2 [Figure 2; (29, 30)]. Apart from these and related chemokines, recent studies have uncovered macrophage axonal guidance by semaphorins, and plexinA (31, 32). While resident macrophage populations, for example in the peritoneal cavity, persist locally, they can be induced by inflammation, to enter lymphatic vessels for delivery to lymph nodes (33), or to enter neighboring organs such as liver, by sterile local injury (34). Blood monocytes of bone marrow origin may remain inside the circulation and interact with the luminal surface of vascular endothelium (35), become part of sinus-lining endothelium, as Kupffer cells, or diapedese into tissues. Such recruited monocytes are transient in blood (24–48 h) and shorter lived (4–7 days) after migration into tissues, compared with resident macrophages of yolk sac origin e.g., microglia, which can be extremely long-lived. Other reservoirs of precursors and mature macrophages are found in splenic red pulp (36) or in secondary haematopoietic organs, such as liver.

While the dual origin of tissue resident macrophages is now widely accepted, there is still uncertainty about the relative contribution of the bone marrow in the adult steady state. In mouse liver, for example, early studies by van Furth and Cohn (37), before their embryonic origin was appreciated, argued for a major contribution of recently dividing bone marrow-derived blood monocytes to resident Kupffer cell populations. The pendulum has swung to yolk sac origin, perhaps too far, as acknowledged by more recent studies (38). The Geissmann group, investigating the origin of murine osteoclasts, showed that after initial perinatal formation of multinucleated cells in bone, monocytes of bone marrow origin are recruited and continue to fuse with osteoclasts throughout adult life (39).

## Growth and Differentiation

Studies by Metcalf (Figure 1) on colony forming cells and lineage-specific growth factors contributed greatly to our understanding of haematopoietic stem cell growth and differentiation *in vitro* (40). Lineage tracing by several groups (41–43) built on studies by Stanley on CSF-1 [reviewed by Chitu and Stanley (44)] and on GM-CSF (45), the major growth/differentiation factors for monocytes, macrophages, and DC. After initial description by von Kolliker in 1873 (Figure 1) (46), Loutit (47) produced proof of the bone marrow origin of osteoclasts; CSF-1 –deficient osteopetrotic op/op mice (48) lacked many, but not all tissue macrophage populations (49). Residual tissue macrophage populations such as microglia, for example, may depend on IL-34, a second ligand for the CSF-1 Receptor, since patients with profound human CSF-1 R deficiency have grossly abnormal CNS development attributed to the absence of microglia (50). Collin et al. have identified mutations which affect monocyte and DC growth and differentiation in humans; bone marrow transplantation and adoptive transfer of haematopoietic stem cells provide clinical and experimental models of monocytopoietic differentiation *in vivo* (51). Recent studies by Olsson et al. (52) and Yanez et al. (53) have demonstrated a binary origin of monocytes in the mouse, exploiting single cell and population RNA seq analysis and adoptive cell transfer.

In spite of these basic discoveries, we need more quantitative information on the number of monocytes, macrophages, and DC in human tissues, and their life span *in vivo*. Yona et al. traced the relationship of human monocytes in the steady state and the kinetic response of monocyte subpopulations to endotoxin administration *in vivo* (54). The subset of monocytic precursors which gives rise to osteoclasts remains to be determined; osteoclasts can be readily produced *in vitro* by culture of monocytes in CSF-1, and Rank Ligand (46), which should facilitate such studies.

## Tissue Distribution and Organ-Specific Properties

In the mouse, we have benefited from the availability of monoclonal antibody markers such as F4/80 to detect macrophages in the developing embryo, in the adult steady state and following a wide range of models of inflammation, infection, malignancy, and atherosclerosis. In addition, we used a panel of mab to identify tissue-specific heterogeneity of marker expression (3). Figure 2 provides a schematic cartoon of these and additional macrophage plasma membrane receptors (55). With the aid of these reagents we identified substantial morphologic and antigenic heterogeneity of resident murine macrophages in different tissue environments such as CNS, spleen and bone marrow (28). Further studies demonstrated heterogeneous antigen expression of monocyte-derived macrophages in BCG-induced granulomata (56), as well as in multinucleated macrophage giant cells (57) and osteoclasts (58). Knowledge of the *in situ* phenotypes of human tissue macrophages is still limited.

## Heterogeneity of Tissue Macrophages: Antigen Markers

The F4/80 antigen(EMR1/ADGRE1), discovered by Austyn and Gordon (59), was used by Hume and others (60) to define monocytes, and macrophages in the mouse. F4/80 is mainly expressed on the plasma membrane, with minimal endocytosis, and is stable to aldehyde fixation; immunocytochemistry therefore can provide exquisite detail of plasma membrane processes in tissue macrophages, suggestive of potential interactions with neighboring cells. Regional variation in morphology and dendritic processes is particularly notable in the brain (61). F4/80, a member of a leukocyte 7-transmembrane, adhesion G protein-coupled receptor family, has been implicated in peripheral tolerance (62), but natural ligands have not been identified. It is also expressed by eosinophils in mouse and human; EMR1 has been identified in other species (63), but expression is transient in human monocyte-derived macrophages. A related molecule, EMR2 (CD312), discovered by Lin and Stacey (64), is expressed by human myeloid cells in blood and tissues, binds chondroitin sulfate B/dermatan sulfate and has been implicated in a human genetic syndrome, vibratory urticaria (65), associated with mast cell degranulation. EMR2 undergoes autoproteolytic cleavage of its extracellular domain to generate an N-terminal polypeptide agonist of GPCR activation.

The F4/80 antigen is expressed during mouse development from midgestation (19) and has been particularly useful in studies of microglia (61). It is also well-expressed in the adult mouse on resident tissue macrophages in the peritoneal cavity, red pulp of spleen, epidermal Langerhans cells, lamina propria of the gut, and Kupffer cells; expression is low on alveolar macrophages in lung, and absent or minimal in white pulp and T-cell rich areas. F4/80 is absent on osteoclasts, metallophilic macrophages in the splenic marginal zone and on subcapsular sinus macrophages in lymph nodes, which express the pan-macrophage endo/lysosomal marker, CD68. Bone marrow- derived monocytes and tissue macrophages recruited to sites of inflammation, infection and malignancy in the mouse express F4/80 strongly.

SIGLEC-1(CD169, sialoadhesin)is a macrophage-specific sialic acid-recognition lectin discovered by Crocker on bone marrow stromal macrophages, at the center of haematopoietic islands (66). It is strongly expressed by marginal metallophils in mouse spleen and by subcapsular sinusoidal macrophages in lymph nodes. It has been implicated in retention and release of monocytes from bone marrow into the circulation. Other lectins widely expressed by macrophages, especially after alternative activation by IL-4/-13, include the macrophage mannose receptor (CD206) (67), and Dectin-1 (CLEC7A), identified as a receptor for fungal beta –glucan by Brown and Gordon (68) and Taylor et al. (3). Scavenger receptors implicated in clearance of apoptotic cells (69), non-opsonic microbial phagocytosis and lipoprotein endocytosis (70), include SRA-I/II, constitutively present on many tissue macrophages (71) and the structurally related collagenous receptor, MARCO (72), which is constitutively expressed by macrophages in the outer marginal zone of rodent spleen (73), but is induced on many tissue macrophages by microbial Toll-like receptor stimulation.

In addition to the above antigens, macrophages express plasma membrane receptors (28) involved in opsonic recognition of IgG antibodies (FcR), complement components (e.g., CD 11b/18), and other opsonins such as milk fat globulin. Other adhesion molecules include various integrins and CD44; plasma membrane receptors that mediate apoptotic cell clearance include an adhesion GPCR BAI-1 (17) and immunoreceptor tyrosine-based activation motif (ITAM) receptors, Tyro, Axl, and MerTK (74). Immunoregulatory receptors include TREM 1 and 2, SIRP alpha, and PD-1. These and receptors for growth factors, cytokines and chemokines have served as useful reagents for FACS, lineage and functional analysis, contributing to our knowledge of macrophage heterogeneity in mouse and human. CD11b expression, for example, is well-expressed on microglia and peritoneal macrophages whereas it is downregulated on alveolar macrophages and Kupffer cells *in situ*.

## Gene Expression

**Figure 3:**
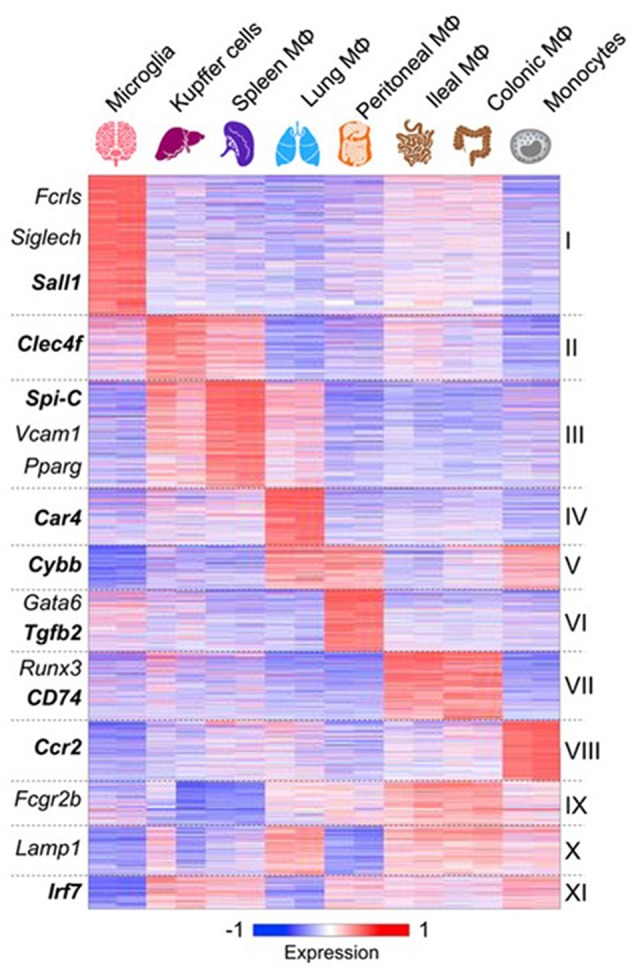
Macrophages express canonical and tissue-specific mRNA signatures. From (77) for further details, with permission. See also (78), ImmGen Consortium (79).

Advances in analysis of macrophage mRNA expression by bulk and single cell sequencing have begun to provide a great deal of new information which has not yet been fully validated by protein expression *in situ* (75–77). However, important conclusions can already be drawn. These studies confirm that macrophages from different tissues are biosynthetically highly active, expressing a large number of diverse, yet canonical macrophage genes. However, tissue macrophages from different organs also express distinctive antigen and mRNA signatures (77) (Figure 3). Recent publications have reported scRNA-seq analysis of blood mononuclear cells (80), embryonic and adult cell populations, including human placenta (81, 82), which contains both fetal, and maternal macrophages. Improved methods of *in situ* protein expression (83, 84) are required to validate heterogeneity of genomic and epigenomic expression by macrophages isolated from different tissues. Spatial reconstruction of immune niches has been proposed by combining photoactivatable reporters and sc RNA-seq(NICHE-seq) (85). Consortia of investigators are contributing to a human tissue atlas (86), which has already lead to discovery of novel cell types and functions. Open access to data will extend knowledge of variation in gene expression by macrophages from different sources. This will illustrate developmental, physiologic, and pathologic expression and functions of resident and monocyte-derived macrophages, as well as indicating the cells with which they interact locally. Striking results have already been reported on the overriding effect of phagocytosis of apoptotic cells on gene expression by macrophages in different sites. These have used *in vivo* models in gut (87), for example, and include parabiotic experiments (88). The microbiome of the gut does not only affect the macrophage phenotype in its local microenvironment, but also systemically (89, 90), through release of microbial products.

## Polarization and Plasticity of Macrophages

We used selected membrane markers to examine the phenotype of mouse peritoneal and human monocyte-derived macrophages in culture, following exposure to Th1 and−2 associated cytokines. In the mouse, IL-4, and subsequently IL-13, was shown to enhance expression and function of mannose receptors (CD206), whereas Interferon gamma selectively downregulated this marker (91). Since MHC class II expression was upregulated by both types of cytokine, we termed this process, alternative, and classical activation, respectively. The terminology M2 and M1 was introduced to include other prototypic stimuli such as immune complexes and macrophage expressed-signatures of selected marker genes (92, 93). We found, using a range of *in vitro* and *in vivo* models, that transglutaminase 2 expression, which is not specific for macrophages, was a consistent marker of alternative macrophage activation in humans and mice (94). Subsequent studies by many investigators showed that macrophage polarization involved a spectrum of changes in gene expression (95); to be a useful concept, we proposed that the term alternative activation should be restricted to the prototypical Th-2 cytokines, IL-4, and IL-13 and their common and specific plasma membrane receptors (96). Microarray analysis of macrophage populations using a range of activation and regulatory stimuli, indicates that modules of genes can be identified as signatures to distinguish among different forms of activation. Further analyses of single cell RNA, and protein expression of gene signatures by yolk sac- and bone marrow-derived macrophages and their correlation with distinct functions such as cytotoxicity, and tissue repair, are required to refine polarization in individual organs.

Both classical and alternative macrophage activation can be divided into two distinct phases, an initial priming step by the appropriate cytokine, and completion by a phagocytic or microbial stimulus which induces further changes in gene expression and serves to localize macrophage effector activity. Microbial uptake enhances cytotoxic and pro-inflammatory activity of interferon-primed, classically activated macrophages, whereas uptake of apoptotic cells by IL-4 treated macrophages, enhances anti-inflammatory gene expression by alternatively primed macrophages (97). In experimental models, LPS can induce paradoxical enhancement of JNK activation following Scavenger receptor ligation of IL-4-primed macrophages, suggesting that the outcome will depend on the nature of the phagocytic receptor involved (98).

Priming of macrophages can also induce an adaptive enhancement of microbial phagocytosis and innate immune function. For example, LPS or microbial stimulation upregulates MARCO expression enabling subsequent enhanced uptake of Neisseria meningitidis via this receptor (99, 100). This observation harks back to the earlier studies of Mackaness on macrophage activation by BCG and Listeria monocytogenes, shown to be antigen dependent, but non-specific for the inducing organism (13). Netea et al. have extended this phenomenon, an example of “trained immunity” (101, 102), and have implicated epigenetic mechanisms in its imprinting.

These concepts are important in attempts to reverse polarization, for example of tumor associated macrophages, for potential immunotherapy. Evidence that the macrophage phenotype *in vivo* is plastic and reversible by adoptive transfer to different tissue microenvironments is sketchy. Van de Laar et al. have shown that yolk sac macrophages, fetal liver and adult monocytes efficiently colonize the empty alveolar niche of *Csf2rb*^−/−^ mice, unlike mature liver peritoneal or colon macrophages (103). We have found that once macrophages have differentiated terminally, for example to a resident peritoneal phenotype, they cannot be induced to express adhesion receptors characteristic of other terminally differentiated macrophages such as those found in bone marrow haemopoietic clusters. Furthermore, experiments need to distinguish between changes in cell populations and individual cells. However, the phenomenon of induced pluripotency (104) indicates that transcription factors and chromatin conformation can enable true plasticity and the ability to give rise to embryonic stem cells, able to generate different somatic cell types, including macrophages (105) and microglia (106) *de novo*.

## Generation of Diversity in Tissue Macrophages

The evidence that resident embryo or bone marrow-derived populations of tissue macrophages, distributed throughout organs in the steady state, acquire distinct phenotypes as well as expressing core macrophage properties, raises a fascinating problem of origin of their diversity. The extent of adaptation by monocytes recruited by infection to different tissue environments, for example in granuloma formation, requires further characterization. In order to establish a testable hypothesis to account for the generation of diversity, we have to keep in mind several properties which distinguish macrophages from T and B lymphocytes, in which antigen receptor gene rearrangement and clonal selection have provided unexpected solutions to account for repertoire diversity and antigen specificity. Macrophages express a broader range of receptors than lymphocytes to distinguish foreign, modified-self and self-ligands; these include proteins and peptides, carbohydrates, nucleic acids, and lipids. Macrophage receptors can be viewed as “hard wired,” unlike the more selective, antigen-specific receptors of adaptive lymphocytes. Tissue macrophages are terminally differentiated, capable of only a limited degree of proliferative capacity once they enter tissues. Clonal selection can therefore be ruled out. We do not know the size of the macrophage repertoire, but it must be substantial to accommodate interactions with other cell types within the body, including macrophages themselves, as well as so-called “pattern recognition receptors” for exogenous and endogenous ligands. Many investigators acknowledge that the local tissue as well as exogenous micro-environment must play a specifying role in inducing or selecting expression of a particular constellation of surface receptors and gene products [for example (75, 107)]. In addition, macrophages can recognize a host of intracellular ligands in their cytosolic, biosynthetic, secretory, or endocytic compartments. However, chromatin conformation, transcription factors and enhancers, in addition to epigenetic mechanisms, must also determine the programme of differential gene expression, and modulation of the macrophage phenotype (108–112). T'Jonck et al. have discussed the role of niche signals and transcription factors involved in tissue resident macrophage development in detail (113).

These considerations leave many questions as to how, when and where, and specifically by which intrinsic and environmental mechanisms, diversity is achieved. Surprisingly little consideration has been given to the nature of the diverse ligands in the extracellular matrix of different tissues (114); nor the role of various epithelia, endothelia, mesenchymal, and neuro-endocrine cells, all of which interact with macrophages as a result of their unique migration and organ distribution (83, 84, 115, 116).

## Tissue-Specific Functions of Macrophages

Tissue macrophages express general, prototypic, functions throughout the body which contribute to homeostasis, recognition and responses to intrinsic and external perturbation, restoring physiologic stability, and contributing to repair after injury. In different organs they adapt to different micro-environments with variations on the themes of clearance of particles and soluble ligands, digestion or storage in lysosomes, constitutive, and induced biosynthesis, and secretion. They interact with living or dying cells and microbes, blood and lymph, undergoing metabolic adaptation, and altering adhesion to extracellular matrix as they migrate, through different locations over time. In the process, they may respond to injurious stimuli by autophagy, cell growth or death. Nevertheless, we can already discern remarkable variations in organ-specific functions to which they contribute; these include a central role in haematopoietic turnover, and haem degradation (117, 118), lymphoid trafficking of immune cells (33); mucosal physiology, for instance in the gut (119, 120); remodeling in the CNS (107, 121, 122); neural- adipose tissue metabolism (123), and adipose- sympathetic nervous interactions (124); and electrical activity in the heart (125). Current studies in single cell RNA and protein expression by tissue macrophages will provide more examples of trophic and defense functions, contributing to embryonic development, anatomic, physiologic, and pathologic processes. Returning to our earlier discussion of how such diversity might be generated, it seems likely that encounters with different ligands in their tissue microenvironment can exploit pre-existing or induce novel sensors to activate adaptive changes in transcription and epigenetic modification; this begs the question of the extent and mechanisms of initial tissue-specific receptor diversification. While differentiation can generate a core panel of recognition molecules on and within the macrophage, it may be necessary to postulate further induction, feedback amplification, or selection by as yet unknown somatic gene expression mechanisms. Investigating the details of osteoclast and DC development *in vivo* and *in vitro* may provide further clues to novel molecular mechanisms.

## Conclusions

Recent progress in molecular and cellular biology have brought exciting insights into view, enabling us to characterize monocyte/macrophage heterogeneity *in situ*. Understanding the themes of their functions within multicellular organisms across a range of evolutionary stages will make it possible to discover a unifying pattern extending far beyond innate or adaptive, cellular and humoral immunity. The challenge will be to imagine the properties underlying the genes and molecules which can lead us to such knowledge. Finally, we need to consider the implications of monocyte/macrophage heterogeneity for therapy. Factors to be taken into account for macrophage-directed immunotherapy include the expression of target antigens on distinct subpopulations, the route of administration, risk of off-target effects and species differences. Similarly, for potential adoptive cell therapy, the origin, differentiation, proliferative capacity and activation status have to be defined, as well as genetic compatibility.

## Author Contributions

SG conceived and wrote the manuscript. AP reviewed and edited the manuscript and designed the figures.

### Conflict of Interest Statement

The authors declare that the research was conducted in the absence of any commercial or financial relationships that could be construed as a potential conflict of interest.

## Abbreviations

ADGR: adhesion 7-transmembrane G-protein coupled receptor
BAI-1: brain-specific angiogenesis inhibitor-1
CSF1-R: macrophage colony stimulating factor receptor
DC: Dendritic cells
EMR: epidermal growth factor-like module-containing, mucin-like hormone receptor-like receptor
ITAM: immunoreceptor tyrosine-based activation motif
GM-CSF: granulocyte macrophage colony stimulating factor
GPCR: G-protein coupled receptor
IL: Interleukin
iPSC: induced pluripotent stem cell
MARCO: macrophage receptor with collagenous domain
MHC: major histocompatibility complex
MPS: Mononuclear phagocyte system
NK: natural killer cell
PD-1: Programmed cell death-1
PMN: polymorphonuclear leukocyte
RES: reticulendothelial system
scRNA seq: single cell ribonucleic acid sequencing
Sirp: signal regulatory protein alpha
SR-A: scavenger receptor A
Tie2: Tyrosine-protein kinase receptor for angiopoietins-2
TLR: toll-like receptor
TNF: tumor necrosis factor
TREM-2: Triggering receptor expressed on myeloid cells-2.
